# Comparative Computational Studies of 3,4-Dihydro-2,6-diaryl-4-oxo-pyrimidine-5-carbonitrile Derivatives as Potential Antinociceptive Agents

**DOI:** 10.3390/molecules17010809

**Published:** 2012-01-16

**Authors:** Janaína V. dos Anjos, Rajendra M. Srivastava, João H. Costa-Silva, Luciana Scotti, Marcus T. Scotti, Almir G. Wanderley, Elisa Soares Leite, Sebastião J. de Melo, Francisco J. B. Mendonça Junior

**Affiliations:** 1 Departamento de Química Fundamental, Universidade Federal de Pernambuco, 50740-540, Recife, PE, Brazil; 2 Núcleo de Educação Física e Ciências do Esporte, Universidade Federal de Pernambuco, 55608-680, Vitória de Santo Antão, PE, Brazil; 3 Laboratório de Tecnologia Farmacêutica, Universidade Federal da Paraíba, Campus I, 50740-540, João Pessoa, PB, Brazil; 4 Departamento de Engenharia e Meio Ambiente, Universidade Federal da Paraíba, Campus IV, 58297-000, Rio Tinto, PB, Brazil; 5 Departamento de Fisiologia e Farmacologia, Universidade Federal de Pernambuco, 50670-901, Recife, PE, Brazil; 6 Departamento de Engenharia Química, Universidade Federal de Pernambuco, 50670-901, Recife, PE, Brazil; 7 Departamento de Antibióticos, Universidade Federal de Pernambuco, 50670-901, Recife, PE, Brazil; 8 Departamento de Ciências Biológicas, Universidade Estadual da Paraíba, Campus V, 58058-420, João Pessoa, PB, Brazil

**Keywords:** 4-(3*H*)-pyrimidinones, antinociceptive activity, molecular modeling, density functional theory

## Abstract

In this study, the antinociceptive properties of 3,4-dihydro-2,6-diaryl-4-oxo-pyrimidine-5-carbonitrile derivatives **5a**–**i** at doses of 25 and 50 mg/kg were evaluated in mice, using the abdominal constriction test. Molecular modeling studies were also performed using density functional theory calculations. These data provided information about the electrostatic and ionization potentials and were used to compare the antinociceptive activity of the title compounds. The most active compounds were 3,4-dihydro-2-(4-chlorophenyl)-6-(4-methoxyphenyl)-4-oxo-pyrimidine-5-carbonitrile (**5b**) and 3,4-dihydro-2,6-diphenyl-4-oxo-pyrimidine-5-carbonitrile (**5i**), which inhibited the number of abdominal constrictions, at 50 mg/kg dose, in 88.6% and 88% of the sample, respectively. A preliminary SAR study demonstrated that halogen replacement in the phenyl rings of the compounds under study reduces the antinociceptive activity. DFT calculations showed that there is a high correlation between the ionization potentials and the analgesic properties of the compounds. It was found that compounds with a positive ionization potential (compounds **5b** and **5i**) were found to be the best analgesic drugs in this series.

## 1. Introduction

Pyrimidinone derivatives are well-known for their pharmacological properties [1,2]. These compounds, structurally related to nucleic acids, have been reported to be anticancer [3,4,5,6,7], interferon inducer [8], antiviral [9,10], anti-hypertensive [11,12], hypoglycaemic [13,14], anticonvulsive [15], anti-histaminic [16], analgesic and antiinflammatory drugs [17,18,19,20,21,22,23].

Several methodologies are available for synthesizing this pharmacologically interesting class of heterocycles. Most of them make use of a condensation reaction between a Michael intermediate and amidines [24], guanidines [25], urea [26], thiourea [27], methylisourea and methylisothiourea [9,10], in the presence of organic bases as catalysts.

**Figure 1:**
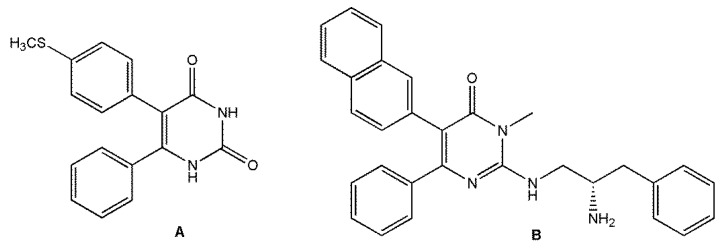
Pyrimidinone derivatives as anti-inflammatory drugs.

As cited above, the pyrimidinone scaffold is present in many analgesic and anti-inflammatory agents [17,18,19,20,21,22,23]. In fact, it has been reported that these substances can modulate the activity of important molecules involved in the inflammatory process, such as COX-2 or protein kinase (p38) modulators [17,18,19,28,29,30,31,32], which could support the pharmacological findings. It is well established that inflammation can stimulate many intracellular signalling pathways, including the p38 pathway, which is considered to be a central regulator of inflammation. For example, COX-2 is a mediator regulated by p38 protein kinase. Regulation of the p38 pathway thus leads to control of the inflammatory mediators and inhibition of the inflammation process [30,31,32]. Figure 1 shows the structures of two pyrimidinone derivatives capable of modulating the inflammatory response, where **A** is considered a COX-2 selective antagonist and **B** is a p38 pathway inhibitor [28,29,30,31,32].

Because of the aforementioned pharmacological potential of this class of compounds, our group has been involved in synthesizing and testing the pharmacological activities of pyrimidinone and pyrimidine derivatives, for the purposes of drug discovery and development [17,18,19,24,27,33,34,35]. Recently, we have synthesized and evaluated the acute toxicity, and anti-edematogenic and antinociceptive activities of 3,4-dihydro-2-phenyl-6-*para*-fluorophenyl-4-oxo-pyrimidine-5-carbo-nitrile, a prototype for this class of compounds, which has shown promising pharmacological activities and low toxicity [19].

The present study thus assesses the antinociceptive activity of a series of previously synthesized pyrimidinones using the abdominal constrictions test. Since the pyrimidinone ring exhibits good analgesic activity, our goal was to produce pharmacologically superior compounds by making changes in the scaffold. In total, nine molecules containing the pyrimidinone ring were tested. We also carried out DFT calculations in order to associate the antinociceptive activity of these compounds with their electronic surfaces through molecular modelling tools.

## 2. Results and Discussion

### 2.1. Antinociceptive Activity

The results regarding antinociceptive activity are displayed in Table 1. It can be seen that the most active compounds are **5b** and **5i**, at the dose of 50 mg/kg, and that the antinociceptive properties of some pyrimidinone derivatives (compounds **5b**,**d**,**i**) are as good as or even better than the reference drug (indomethacin).

In this study, antinociceptive activity was evaluated using acetic acid induced stimuli. The acetic acid writhing test is a peripheral and visceral nociception model, consisting of high intensity stimuli and rapid nociceptive response [36]. Acetic acid-induced effects can be eliminated using a wide range of analgesics, including non-steroidal anti-inflammatory drugs (such as indomethacin). Most non-steroidal anti-inflammatory drugs act as non-selective antagonists of the enzyme, cyclooxygenase, inhibiting both the cyclooxygenase-1 and cyclooxygenase-2 isoforms. These enzymes catalyze the production of inflammatory messengers from arachidonic acid and these mediators can play the role of messengers in the inflammation process.

Almost all drugs, at 25 and 50 mg/kg doses, significantly reduced the number of abdominal constrictions. It can also be seen, in all cases, that the analgesic effect was dose-dependent. Earlier studies have shown that this type of nucleus shows anti-inflammatory activity in animal models [17,18,19,20,21,22,23,28,29]. In view of these results and a comparison with the results found in earlier studies [17,18,19,28,29], we suggest that pyrimidinone compounds can interfere with the acute inflammatory response induced by acetic acid. Therefore, the title compounds might inhibit or even modulate the migration or production of chemical mediators in the inflammatory site [37]. The compounds described here may act as COX inhibitors, as described earlier in the literature for other pyrimidinone compounds [17,18,19,28,29]. However, from the data obtained in the present study, we are not able to determine the exact mechanism involved in the antinociceptive effects produced by these compounds. More specific and detailed studies therefore need to be carried out to better understand the pharmacological mechanisms involved.

It can be seen that the most active compounds are **5b** and **5i**, which exhibited more than 88% of antinociceptive activity in relation to the negative control group. These numbers are as significant as those observed for indomethacin (approximately 76%). In contrast to these findings, we found that compound **5h** was the least active of this series. These results indicate that the replacement of hydrogen atoms in the phenyl rings by fluorine atoms decreases the biological activity (**5i** against **5h**). This can be extended to **5f**, which was less active than **5i**, but better than **5h**. This clearly demonstrates that the replacement of hydrogen atoms by fluorine atoms in both phenyl rings is even more prejudicial than introducing a fluorine atom into one ring. In the halogen-containing compounds, a chlorine substitution for a hydrogen atom seems to be less harmful than fluorine substitution in terms of antinociceptive activity (**5e** against **5f** and **5b** against **5g**). The other interesting feature is that the electron-donating groups (EDG), such as the methoxy group at R^1^, do not alter these properties (**5d** against **5i** and **5b** against **5d**). However, the compound becomes less active when R^2^ = OCH_3_ (**5a** and **5c** against **5d**).

### 2.2. Molecular Modeling Studies

**Figure 2:**
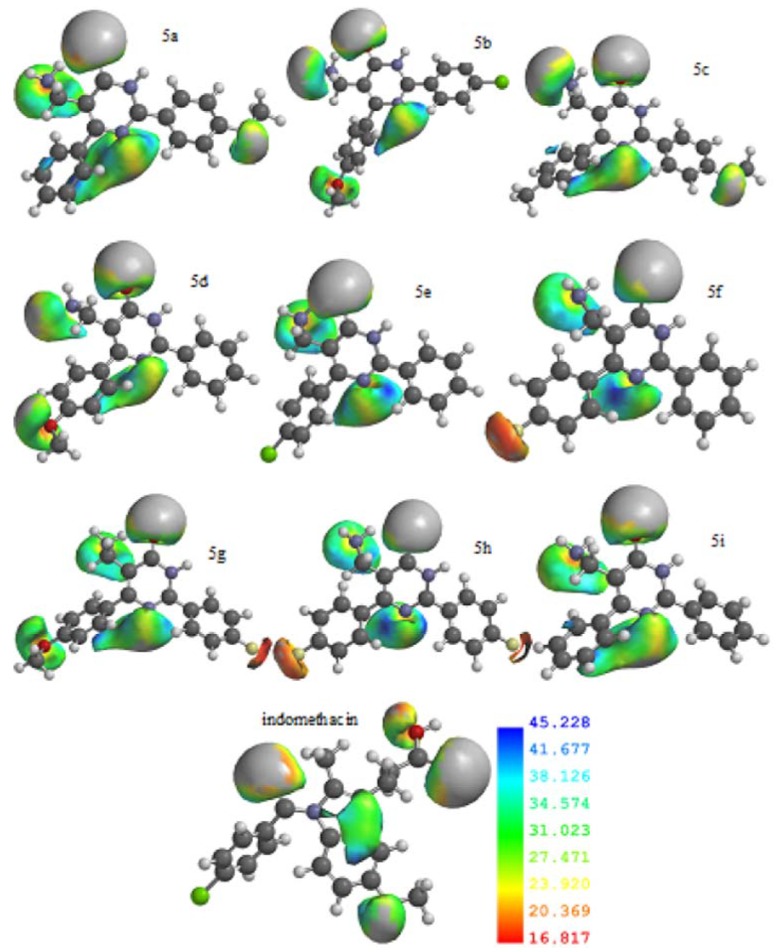
Comparative local ionization potentials of compounds **5a**–**i** and indomethacin.

It was confirmed that the ionization potential is closely related to the antinociceptive activity exhibited by the pyrimidinone derivatives. Of the electronic surfaces calculated here using DFT, the ionization potential showed the greatest significant differences between the most and least active compounds. These calculations for the local ionization potentials of compounds **5a**–**i** can be visualized in the tube model representation (Figure 2). The atoms are represented by colors: carbon (gray), nitrogen (blue), oxygen (red), chlorine (green), fluorine (yellow) and hydrogen (white).

We found that compounds **5f**, **5g** and **5h** show negative ionization potential, (orange-red regions), with **5h** being the less active compound. On the other hand, good antinociceptive compounds, particularly **5b** and **5i**, have positive ionization potentials (blue-green regions), similar to the reference drug (indomethacin). This can be clearly seen in **5b**, the most active compound.

This simple molecular modeling study showed how this calculation tool can be useful in predicting the particular activity of an investigative series of compounds. In conclusion, DFT calculations showed that there is a high correlation between the ionization potential and the biological activity exhibited by the title compounds.

## 3. Experimental 

### 3.1. 3,4-Dihydro-2,6-diaryl-4-oxo-pyrimidine-5-carbonitriles

**Scheme 1:**
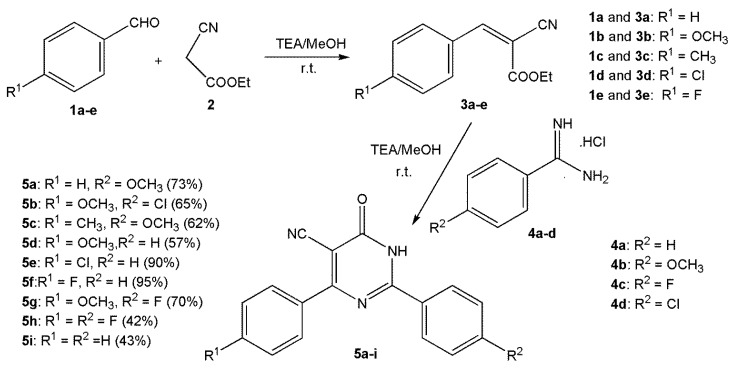
Synthesis of compounds **5a**–**i**.

Compounds **5a**–**i** used in this study were synthesized earlier by our research group [24]. A schema showing the synthesis of this series of compounds is shown below (Scheme 1). Indomethacin (reference drug) was purchased from Sigma-Aldrich.

### 3.2. Animals

Swiss male mice weighing 30 ± 5 g were used for pharmacological assays. They were kept under standard environmental conditions (22 ± 2 °C; 12:12 h dark/light cycle). Water and food (Labina^®^, Purina, Brazil) were provided *ad libitum*. The experimental protocols were approved by the Animal Experimentation Ethics Committee of the Universidade Federal de Pernambuco, UFPE (Process n° 015683/2005-34) and are in accordance with the National Institute of Health Guidelines for the Care and Use of Laboratory Animals.

### 3.3. Antinociceptive Activity

The pharmacological tests were preceded by acute toxicity assays in mice in order to determine the doses to be used in this work [19]. The abdominal constrictions test was used to assess the antinociceptive activity of the synthesized compounds [38,39]. In this test, abdominal wall muscle constrictions and elongations, often followed by a characteristic hind-limb extension, are induced by acetic acid. The animals were kept without food overnight and divided in groups of six animals. The pyrimidinone derivatives were individually dissolved in a prepared 2.5% Tween 80 solution diluted in NaCl 0.9%. The drugs (compounds **5a**–**i**) were given in the corresponding dose (25 or 50 mg/kg) intraperitoneally. After 15 minutes, the animals received the 0.8% acetic acid solution (1 mL/kg, intraperitoneally) and the writhes were counted for 30 minutes. The negative control group received the vehicle (2.5% Tween 80 in NaCl 0.9%), whereas the positive control group received indomethacin (10 mg/kg) before injection of acetic acid. The results were expressed as the reduction in the number of abdominal constrictions in relation to the negative control group.

### 3.4. Computational Methods

The Hyperchem v. 8.0 program [40] was used to draw the chemical structures of the compounds under study and their geometry optimized using an MM+ force field [41]. A new geometry optimization was then performed using the semi-empirical method AM1 (Austin Model 1) [42]. The optimized structures were subjected to conformational analysis using the random search method [43,44] and the options for 1,000 interactions and 100 cycles of optimization, and 10 was established as the number of conformers of lowest minimum energy. The selected dihedrals were evaluated in rotation in accordance with the standard conditions (default) of the program, *i.e.*, number of simultaneous variations from 1 to 8; acyclic chains were submitted to rotations from 60 to 180° and ring torsions in the range of 30 to 120°.

The conformer of lowest minimum energy was selected, saved as .mol and exported to the Spartan 8 program [45]. The electrostatic potential, ionization potential and the HOMO and LUMO orbital surface of all compounds were obtained. To obtain and compare the surfaces of the ionization potential we adopted the range from 16,000 (red) to 45,000 (blue), and for comparison of the electrostatic potential maps, −284,000 (red) to 164,000 (blue).

## 4. Conclusions

All pyrimidinone derivatives tested presented good antinociceptive activity, with a dose-dependent pattern. The most active compounds were **5b** and **5i**, at the dose of 50 mg/kg of body weight. Our results have shown that replacement of the hydrogen atom by fluorine in the phenyl rings of 3,4-dihydro-2,6-diaryl-4-oxo-pyrimidine-5-carbonitriles causes a reduction in analgesic activity. The comparative study of molecular modeling corroborated pharmacological trials, showing a high correlation between ionization potential and analgesic activity. The compounds that showed positive ionization potential (*i.e*., **5b** and **5i**) were also the most active in the antinociceptive activity tests.

## Figures and Tables

**Table 1:** Antinociceptive activity of **5a**–**i** and indomethacin in the acetic acid writhing test. Data are expressed as the mean ± SD for the dose-response profile of six animals.

Compd.	Dose (mg/kg)	Antinociceptive activity (%)
**5a**	25	-
	50	71.5 ± 6.9
**5b**	25	49.5 ± 9.9
	50	88.6 ± 3.4
**5c**	25	-
	50	75.1 ± 5.8
**5d**	25	68.8 ± 8.2
	50	86.0 ± 4.1
**5e**	25	56.4 ± 11.9
	50	73.9 ± 10.0
**5f**	25	22.1 ± 16.1
	50	71.4 ± 8.5
**5g**	25	57.7 ± 9.9
	50	70.0 ± 5.4
**5h**	25	47.4 ± 13.0
	50	61.0 ± 13.7
**5i**	25	85.4 ± 4.6
	50	88.0 ± 4.0
**Indomethacin**	10	76.3 ± 4.8
